# Evaluation of patients with respiratory infections during the first pandemic wave in Germany: characteristics of COVID-19 versus non-COVID-19 patients

**DOI:** 10.1186/s12879-021-05829-x

**Published:** 2021-02-10

**Authors:** Nicola Fink, Johannes Rueckel, Sophia Kaestle, Vincent Schwarze, Eva Gresser, Boj Hoppe, Jan Rudolph, Sophia Goller, Wolfgang G. Kunz, Jens Ricke, Bastian O. Sabel

**Affiliations:** 1 Department of Radiology, University Hospital, LMU Munich, 81377 Munich, Germany; 2 Comprehensive Pneumology Center (CPC-M), German Center for Lung Research (DZL), 81377 Munich, Germany

**Keywords:** Respiratory infection, COVID-19, SARS-CoV2, Characteristics, Pneumonia, Laboratory parameters, Computed tomography

## Abstract

**Background:**

Characteristics of COVID-19 patients have mainly been reported within confirmed COVID-19 cohorts. By analyzing patients with respiratory infections in the emergency department during the first pandemic wave, we aim to assess differences in the characteristics of COVID-19 vs. Non-COVID-19 patients. This is particularly important regarding the second COVID-19 wave and the approaching influenza season.

**Methods:**

We prospectively included 219 patients with suspected COVID-19 who received radiological imaging and RT-PCR for SARS-CoV-2. Demographic, clinical and laboratory parameters as well as RT-PCR results were used for subgroup analysis. Imaging data were reassessed using the following scoring system: 0 – not typical, 1 – possible, 2 – highly suspicious for COVID-19.

**Results:**

COVID-19 was diagnosed in 72 (32,9%) patients. In three of them (4,2%) the initial RT-PCR was negative while initial CT scan revealed pneumonic findings. 111 (50,7%) patients, 61 of them (55,0%) COVID-19 positive, had evidence of pneumonia. Patients with COVID-19 pneumonia showed higher body temperature (37,7 ± 0,1 vs. 37,1 ± 0,1 °C; *p* = 0.0001) and LDH values (386,3 ± 27,1 vs. 310,4 ± 17,5 U/l; *p* = 0.012) as well as lower leukocytes (7,6 ± 0,5 vs. 10,1 ± 0,6G/l; *p* = 0.0003) than patients with other pneumonia. Among abnormal CT findings in COVID-19 patients, 57 (93,4%) were evaluated as highly suspicious or possible for COVID-19. In patients with negative RT-PCR and pneumonia, another third was evaluated as highly suspicious or possible for COVID-19 (14 out of 50; 28,0%). The sensitivity in the detection of patients requiring isolation was higher with initial chest CT than with initial RT-PCR (90,4% vs. 79,5%).

**Conclusions:**

COVID-19 patients show typical clinical, laboratory and imaging parameters which enable a sensitive detection of patients who demand isolation measures due to COVID-19.

## Background

In December 2019 an outbreak of a new coronavirus occurred in Wuhan, China [1]. This virus was designated as “Severe Acute Respiratory Syndrome Coronavirus 2” (SARS-CoV-2) [2]. The associated disease was defined as “coronavirus disease 19” (COVID-19) [3]. Within no time SARS-CoV-2 spread across the globe and was finally declared a pandemic in March 2020 [4]. Within Germany, Bavaria and especially Munich are among the most severely and first affected regions.

COVID-19 usually manifests with symptoms of a respiratory disease including, above all, fever and cough [5–9]. Several studies have revealed typical laboratory characteristics in COVID-19 patients, such as an increase of C-reactive protein (CRP) and lactate dehydrogenase (LDH) as well as leukocytopenia [5, 10, 11], and typical findings in chest computed tomography (CT), including bilateral, multilobar ground glass opacities and/or consolidations especially in the peripheral lung zones [12–16]. Currently, the most established method to detect SARS-CoV-2 is the real-time reverse transcription polymerase chain reaction (RT-PCR) [17, 18]. However, several studies showed limitations of this testing method regarding its sensitivity: While in some COVID-19 patients the initial RT-PCR was negative, the initial CT scan already revealed typical findings [19, 20]. This suggests a higher sensitivity of CT scans for the detection of SARS-CoV-2. In addition, RT-PCR requires a long time until results are available, whereas chest CT is performed and assessed within a few minutes. In clinical practice, prevailing conditions such as limited laboratory capacities and high patient numbers can delay the results of RT-PCR, even though the analysis itself takes only a few hours. The delay in decision-making on isolation and appropriate treatment or in some cases even the missing detection of COVID-19 due to low sensitivity of RT-PCR ties up health care resources and implies a risk with regard to the further spread of the virus. Thus, the SARS-CoV-2 pandemic is also challenging radiological institutes. The analysis of COVID-19 typical clinical and radiological characteristics is crucial in order to identify its potential for efficient patient management. So far, most studies analyzed cohorts including patients with confirmed COVID-19 [5–9, 11, 21]. Only a few studies compared patients with and without COVID-19 in a cohort with respiratory infections suspicious for COVID-19, but mainly with a small sample size [13, 22, 23], with a low rate of confirmed COVID-19 cases [24] or without analysis of radiological imaging [25]. Nevertheless, it is precisely this collective of cases with suspected COVID-19 that occurs in emergency departments during the pandemic and ties up health care resources to a large extent. It is therefore crucial to analyze the characteristics of COVID-19 patients in such a cohort in order to transfer the results to everyday clinical practice in emergency departments.

Therefore, this prospective and single-center study aimed to define clinical and radiological characteristics of COVID-19 patients within a cohort with respiratory infections in the emergency department of one of Germany’s largest university hospitals, situated in one of the most affected regions in Germany. We hypothesize that there are important differences between COVID-19 positive and negative patients with a respiratory infection regarding these characteristics. This may be a useful adjunct to enable a sensitive and early detection of COVID-19 in emergency departments, also with regard to isolation of patients, which is mandatory to prevent an ongoing viral spread but on the other hand ties up a relevant proportion of healthcare capacities.

## Methods

### Patient population and data sources

We prospectively included 219 patients who were presented in the emergency department of the University Hospital, LMU Munich, from March 16 to April 12 2020 with signs of a respiratory infection suspicious for COVID-19 and received radiological imaging (chest radiographs/CXR and / or CT) as well as RT-PCR for SARS-CoV-2. Patients were defined as COVID-19 positive depending on the result of RT-PCR. The initial RT-PCR was mainly done with samples of nasopharyngeal and oropharyngeal swab as standard in the emergency department. In the case of suspected COVID-19 despite negative initial RT-PCR, analysis of lower respiratory specimens such as endotracheal aspirate and bronchoalveolar lavage was performed in some cases (24 out of 57 patients with repeated testing despite initially negative result; 42,1%). Patient age and gender were recorded in all included patients, chronic comorbidities were additionally registered in COVID-19 positive patients. Comorbidities were classified into the following groups: respiratory, cardiovascular, oncological, neurological, presence of diabetes mellitus and others. In case of admission, overall duration of hospitalization and intensive care unit (ICU) stay was recorded. Furthermore, duration of symptoms, initial body temperature and relevant initial laboratory values (CRP, leukocytes, interleukin-6/IL-6 and LDH) at the time of presentation were recorded. Regarding IL-6 values, one outliner in the Non-COVID-19 cohort with a value of 75,034 pg/ml (threshold: ≤ 5,9 pg/ml) was excluded from the statistical analysis.

### Image acquisition

Radiological imaging of the chest included CXR and CT scans at the time of presentation. CXRs were obtained in upright or supine position at full inspiration. CT scans were performed as native high-resolution or contrast-enhanced (in case of suspected pulmonal embolism) CT scan (Somatom Force [Siemens Healthineers / Erlangen / Germany], Somatom Definition AS+ [Siemens Healthineers / Erlangen / Germany] and Optima 660 [GE Healthcare / Chalfont St Giles / Great Britain]).

### Image interpretation / radiologist annotation

One board-certified radiologist as well as two radiology residents (with 2 / 3 years of experience in thoracic imaging) evaluated the CXRs and CT scans by consensus regarding pneumonic features and COVID-19 typical findings. Readers were blinded to RT-PCR results as well as clinical and laboratory data. In CXR images the presence of pneumonic features was rated using the following scoring system: 0 – absent, 1 – possible and 2 – present. CT scans were classified according to two different reading scores: 1) presence of pneumonic features (0 – absent, 1 – present) and 2) presence of COVID-19 typical features (0 – not typical, 1 – possible, 2 – highly suspicious). According to the current literature, COVID-19 typical features were defined as ground glass opacities (GGO) with or without “crazy paving” and/or consolidations with peripheral emphasis [26]. In addition, pneumonic findings in CT scan were evaluated by radiological readers per lung lobe regarding the presence of ground glass opacities and/or consolidations.

### Statistical analysis

Subgroups were defined based on the result of RT-PCR for SARS-CoV-2 and the CT findings regarding the presence pneumonic features. Comparison of those subgroups was done by Fisher’s exact test. Results were graphically illustrated using the software GraphPad Prism (Version 8.4.2, GraphPad, San Diego, California, USA). Continuous variables were statistical analyzed using Mann-Whitney U-Test. The sensitivity, specificity, positive predictive value (PPV), negative predictive value (NPV) and accuracy of initial RT-PCR and initial chest CT for the detection of patients requiring isolation were calculated using overall RT-PCR and CT scan in a sensitive reading for COVID-19 (score 1 and 2) as reference.

## Results

### Study population and subgroup definition

**Fig. 1:**
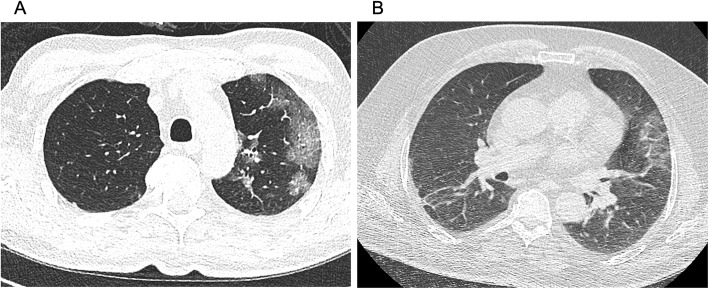
Two COVID-19 patients with initially negative RT-PCR, but highly suspicious findings in initial CT scan. Patient A was tested positive for COVID-19 in the second RT-PCR and patient B in the fourth, while initial CT scan already revealed typical findings

**Fig. 2:**
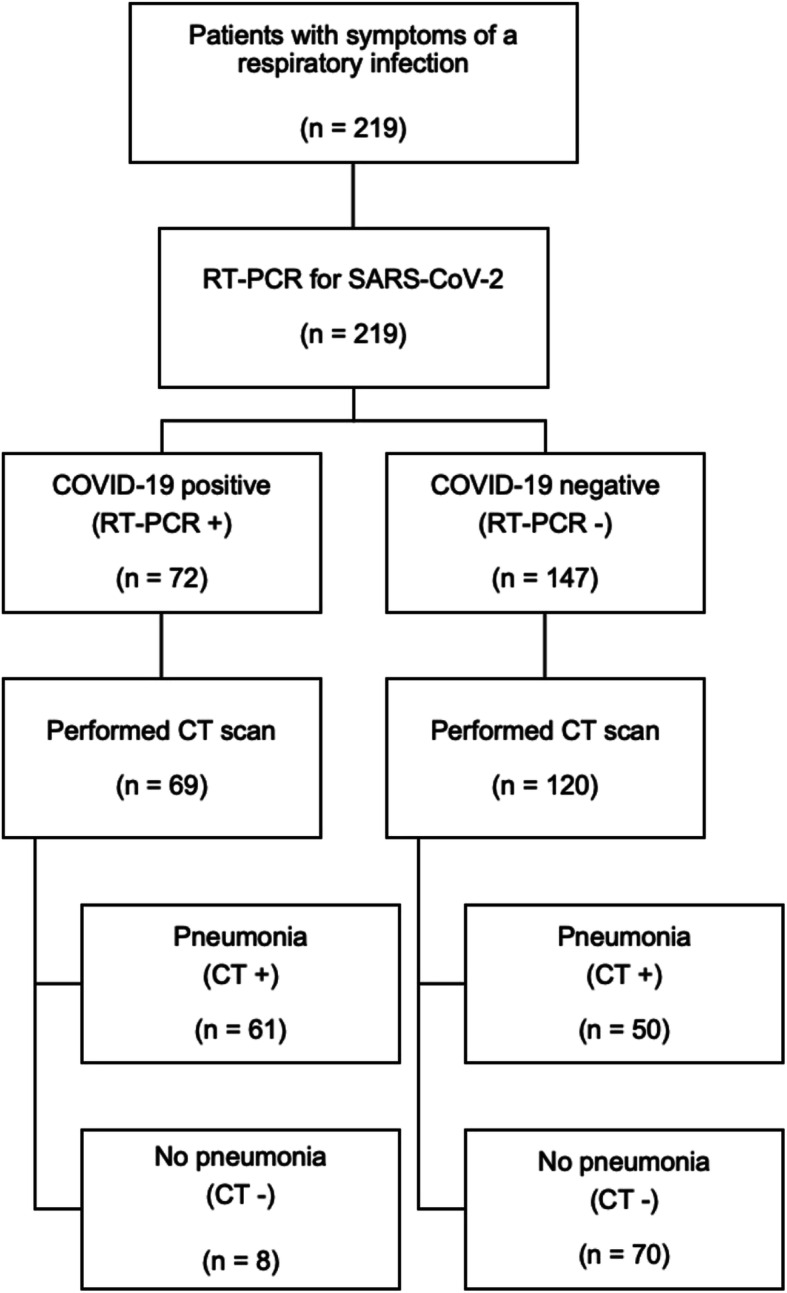
Flowchart illustrating the composition of our study population and subgroups

In total, 219 patients with suspected COVID-19 due to respiratory infection were presented at the emergency department of our university hospital within 4 weeks and received radiological imaging of the chest (CT or CXR) as well as SARS-CoV-2 testing by RT-PCR. The average age was 59.6 ± 1.3 (range: 19–99) years, gender distribution 132 men and 87 women. 72 (32.9%) patients were tested positive for SARS-CoV-2 by RT-PCR and therefore defined as COVID-19 positive, 147 (67.1%) were tested negative and defined as COVID-19 negative. In the COVID-19 negative subgroup, 52 (35,4%) patients were tested negative repeatedly, including 34 (23,1%) with two, 16 (10,9%) with three, and two with four (2,7%) negative tests for SARS-CoV-2. Among patients with positive results, the initial test was negative in three cases (4.2%), only further tests turned out to be positive: one patient had COVID-19 confirmed by two tests, one patient by three tests and one patient by four tests. In all of them the initial CT scan showed pneumonic findings. Figure 1 shows the CT scans of two of these patients in whom the changes in initial chest CT were evaluated as highly suspicious for COVID-19 despite initially negative RT-PCR. Subgroups of COVID-19 positive and negative patients did not significantly differ in age and gender distribution. CT scan was performed in 189 patients. Based on the result of RT-PCR and the presence of pneumonic features in chest CT further subgroups were defined (Fig. 2): 1) COVID-19 positive patients with pneumonic findings (PCR +, CT +; 61 out of 189, 32.3%), 2) COVID-19 positive patients without pneumonic findings (PCR +, CT -; 8 out of 189, 4.2%), 3) COVID-19 negative patients with pneumonic findings (PCR -, CT +; 50 out of 189, 26.5%), 4) COVID-19 negative patients without pneumonic findings (PCR -, CT -; 70 out of 189, 37.0%). Subgroups of patients with pneumonic features in CT scan did not significantly differ in age and gender distribution.

**Fig. 3:**
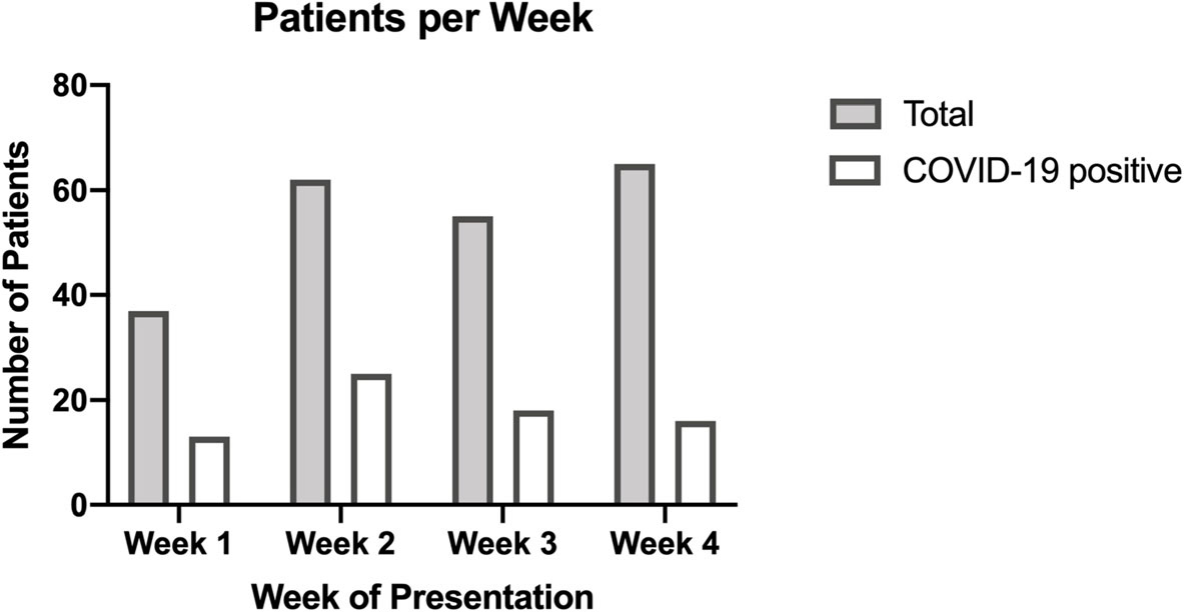
Patients with suspected COVID-19 per week (16 March to 12 April 2020)

In the course of the observed weeks at the beginning of the pandemic in Germany, the number of patients presenting themselves in the emergency department with suspected COVID-19 and receiving radiological imaging showed the following changes (Fig. 3): An increase in the number of patients and detected COVID-19 cases was recorded between the first and the second week (increase of 67.6% presenting patients and 92.3% of COVID-19 cases). In the following weeks the number of presenting patients remained at a similar level, the number of COVID-19 positive patients decreased (decrease of 36.0% from week two to four).

**Fig. 4:**
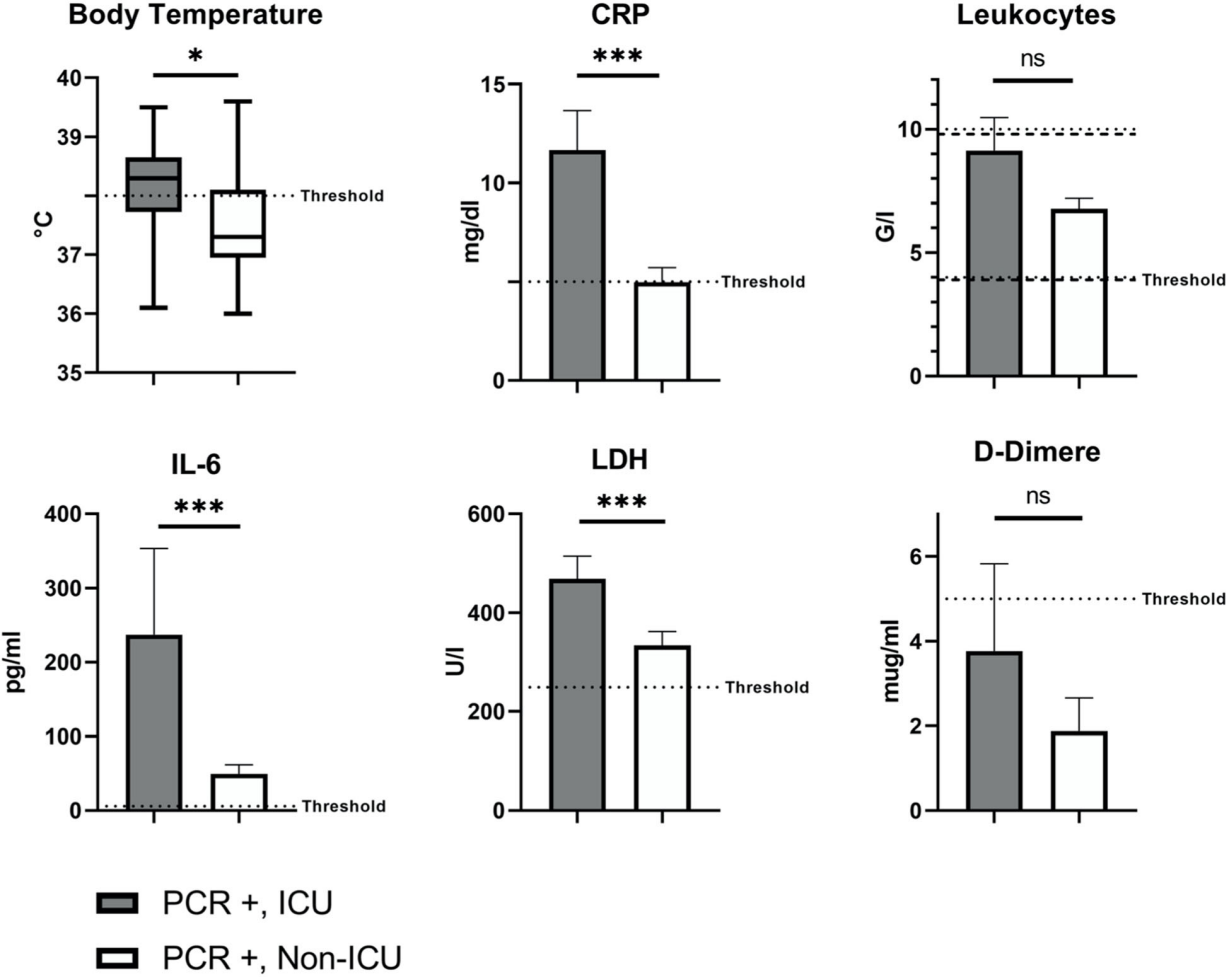
Body temperature and laboratory values compared between ICU and Non-ICU subgroup within confirmed COVID-19 patients. “PCR +” includes COVID-19 patients confirmed by RT-PCR. * *p* < 0.05; ** *p* < 0.01; *** *p* < 0.001; ns = not significant

A total of 174 patients were hospitalized (79.5%) with a higher proportion among the COVID-19 positive patients (67 out of 72 patients, 93.1% vs. 72.8%; *p* = 0.0003). Among COVID-19 positive patients a total of 18 (26.9%) were admitted to an intensive care unit (ICU-COVID-19 subgroup), on average 1.8 ± 0.6 days after hospitalization. Those COVID-19 patients admitted to an ICU had higher body temperature (38.2 ± 0.2 vs. 37.5 ± 0.1 °C; *p* = 0.011; threshold: ≤ 38 °C; Fig. 4) as well as elevated levels of CRP (11.7 ± 2.0 vs. 5.0 ± 0.7 mg/dl; *p* = 0.0003; threshold: ≤ 5 mg/dl; Fig. 4), LDH (468.7 ± 45.5 vs. 334.4 ± 27.9 U/l; *p* = 0.0004; threshold: ≤ 249 U/l; Fig. 4) and IL-6 (237.1 ± 116.2 vs. 49.4 ± 12.6 pg/ml; *p* = 0.0002; threshold: ≤ 5.9 pg/ml; Fig. 4) than COVID-19 patients not admitted to an ICU (Non-ICU-COVID-19 subgroup). Leukocyte counts and D-dimer values did not significantly differ between these subgroups (Fig. 4).

At the time of data collection, 146 patients could already be discharged from hospital after a mean hospitalization time of 8.6 ± 0.5 days with a longer hospitalization of COVID-19 patients (10.4 ± 1.0 vs. 7.5 ± 0.5 days; *p* = 0.019). Three patients with confirmed SARS-CoV-2 died (4.2%).

A detailed overview of patient outcome is given in Table 1*.*

**Table 1:** Clinical outcome of our study cohort

Characteristics n (%) if not labelled differently	COVID-19 positive*		COVID-19 negative*		***p***-value	
	Total (*n =* 72)	With pneumonia in CT (*n* = 61)	Total (*n* = 147)	With pneumonia in CT (*n* = 50)	Total	With Pneumonia
**Clinical course**						
**Hospitalization**	67 (93.1)	60 (98.4)	107 (72.8)	45 (90.0)	**0.0003**	0.089
Duration of hospitalization in days** (mean ± SEM)	10.4 ± 1.0	11.3 ± 1.1	7.5 ± 0.5	9.8 ± 1.0	**0.019**	0.404
**Admission to ICU**	18 (26.9)	18 (30.0)	15 (14.0)	12 (26.7)	**0.047**	0.828
Hospitalization to ICU in days (mean ± SEM)	1.8 ± 0.6	1.8 ± 0.6	1.7 ± 0.8	1.9 ± 0.9	0.474	0.500
Duration of intensive care in days** (mean ± SEM)	10.6 ± 2.3	10.6 ± 2.3	7.3 ± 1.5	8.0 ± 1.6	0.367	0.550
**Outcome**						
Discharged	53 (79.1)	46 (76.7)	93 (86.9)	37 (82.2)		
Still hospitalized***	11 (16.4)	11 (18.3)	11 (10.3)	6 (13.3)		
Dead	3 (4.5)	3 (5.0)	3 (2.8)	2 (4.4)		
**No hospitalization**	5 (6.9)	1 (1.6)	40 (27.2)	5 (10.0)		

*based on the result in RT-PCR for SARS-CoV-2; **number of patients for whom the values were available; **in patients who are already discharged from hospital or at least intensive care unit (ICU); ***on April 27 2020

### Clinical characteristics

**Fig. 5:**
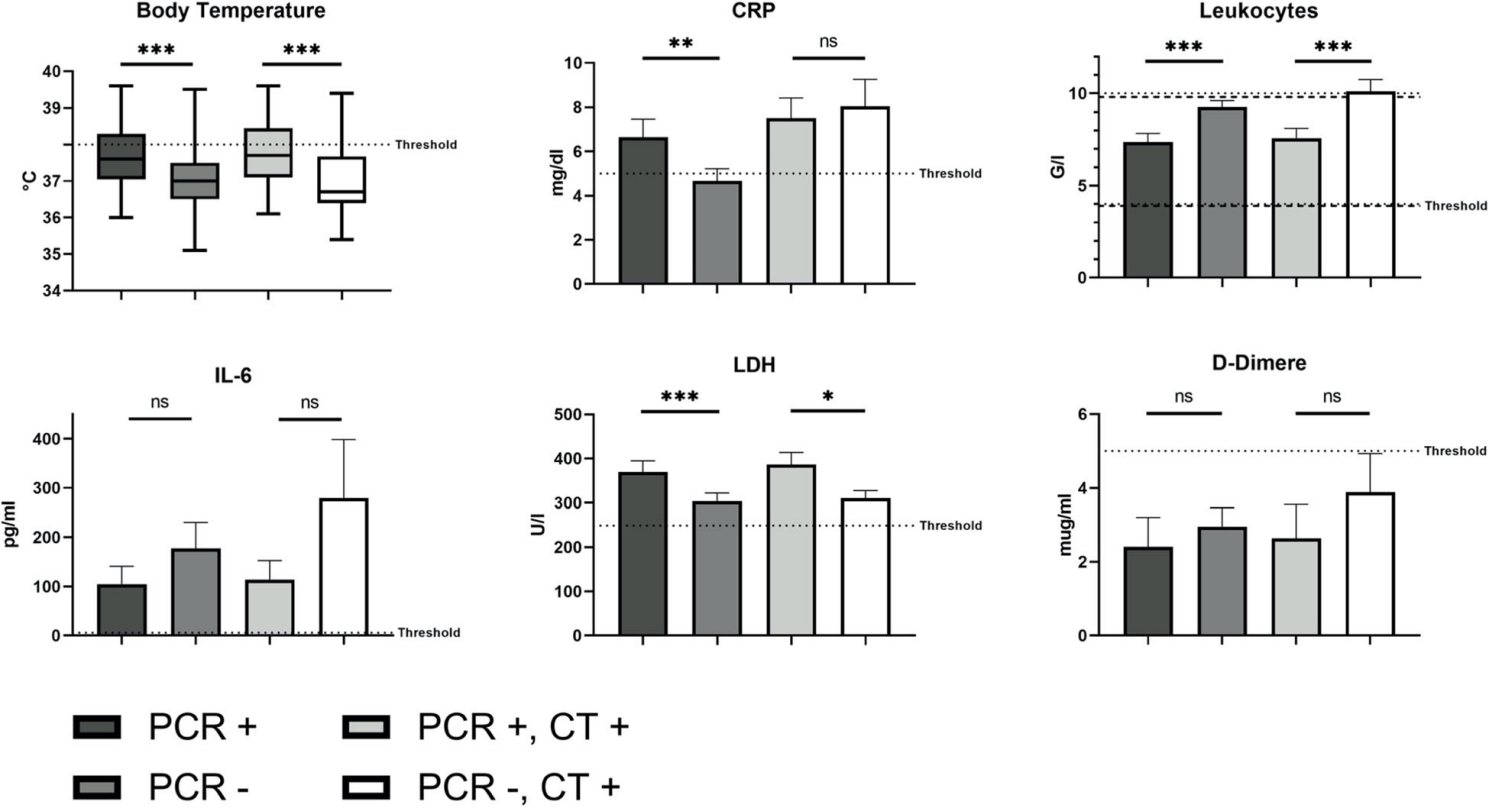
Body temperature and laboratory values: 1) COVID-19 positive vs. negative, 2) COVID-19 pneumonia vs. other pneumonia. “PCR +” includes COVID-19 patients confirmed by RT-PCR. “PCR -“ includes patients with a negative result for SARS-CoV-2 in RT-PCR. “CT +” includes patients with pneumonic findings in chest CT. * *p <* 0.05; ** *p <* 0.01; *** *p <* 0.001; ns = not significant

The duration of symptoms at presentation in the emergency department averaged 6.8 ± 6.3 days with a significantly delayed presentation after onset of symptoms in patients with COVID-19 (7.3 ± 0.7 vs. 6.6 ± 0.8 days; *p* = 0.01). In addition, body temperature was significantly higher in patients with confirmed COVID-19 (37.7 ± 0.1 vs. 37.1 ± 0.1 °C; *p* < 0.0001; threshold: ≤ 38.0 °C; Fig. 5). Fever, defined as a body temperature over 38 °C, was more frequent in the COVID-19 subgroup than in the Non-COVID-19 subgroup (40.0% vs. 15.0%, *p* = 0.0001). However, in case of fever in COVID-19 patients, body temperature was mainly just slightly above the threshold of 38 °C with a mean body temperature of 38,6 ± 0,1 °C in the respective group. Furthermore, patients with confirmed COVID-19 showed significantly higher values of CRP (6.7 ± 0.8 vs. 4.7 ± 0.6 mg/dl, *p* = 0.003; threshold: ≤ 5 mg/dl; Fig. 5) and LDH (370.0 ± 24.7 vs. 303.5 ± 18.6 U/l, *p* < 0.0001; threshold: ≤ 249 U/l; Fig. 5). At the same time, leukocyte counts were significantly lower in the COVID-19 subgroup: 7.4 ± 0.5 vs. 9.3 ± 0.3 G/l (p < 0.0001; threshold: female 4.0–10.4 G/l, male 3.9–9.8 G/l; Fig. 5). Both groups did not significantly differ regarding IL-6 and D-dimer values (Fig. 5).

When considering only patients with pneumonic features in CT scan, the comparison between COVID-19 positive and negative patients confirmed by RT-PCR showed the following results: Patients with COVID-19 pneumonia showed longer symptom duration at presentation in the emergency department (7.3 ± 0.6 vs. 4.9 ± 0.8 days; *p* = 0.003) as well as significantly higher body temperature (37.7 ± 0.1 vs. 37.1 ± 0.1 °C; *p* = 0.0001; threshold: ≤ 38 °C) and LDH values (386.3 ± 27.1 vs. 310.4 ± 17.5 U/l; *p* = 0.012; threshold: ≤ 249 U/l) than patients with pneumonia due to other cause (Fig. 5). At the same time, leukocyte counts were significantly lower in patients with COVID-19 pneumonia (Fig. 5): 7.6 ± 0.5 vs. 10.1 ± 0.6 G/l; *p* = 0.0003 (threshold: female 4.0–10.4 G/l, male 3.9–9.8 G/l). Groups of COVID-19 positive and negative patients with pneumonic features in CT scan did not differ significantly regarding age, sex, CRP, IL-6 and D-dimer values. The initial characteristics are presented in detail in Table 2.

**Table 2:** Demographic, clinical and diagnostic characteristics at initial presentation of our study cohort

Diagnostic features n (%) if not labelled differently	COVID-19 positive^**a**^		COVID-19 negative^**a**^		***p***-value	
	Total (*n =* 72)	With pneumonia in CT (*n* = 61)	Total (*n =* 147)	With pneumonia in CT (*n =* 50)	Total	With Pneumonia
**Age** in years (mean ± SEM)	60.0 ± 2.0	63.1 ± 2.0	59.5 ± 1.7	61.7 ± 2.9	0.955	0.903
**Sex**					0.141	0.534
Male	49 (68.1)	45 (73.8)	83 (56.5)	34 (68.0)		
Female	23 (31.9)	16 (26.2)	64 (43.5)	16 (32.0)		
**Comorbidities in COVID-19 patients**						
Total (≥1 system)	52 (72.2)	46 (75.4)				
Respiratory	10 (13.9)	32 (52.4)				
Cardiovascular	35 (48.6)	9 (14.8)				
Oncological	7 (9.7)	7 (11.5)				
Neurological	8 (11.1)	7 (11.5)				
Diabetes	11 (15.3)	10 (16.4)				
Others	32 (44.4)	28 (45.9)				
**Duration of symptoms** at presentation in days (mean ± SEM)	7.3 ± 0.7 (*n* = 52)**	7.3 ± 0.6 (*n* = 45)**	6.6 ± 0.8 (*n* = 85)**	4.9 ± 0.8 (*n* = 29)**	**0.010**	**0.003**
**Body temperature** at presentation in °C (mean ± SEM)	37.7 ± 0.1 (*n* = 65)**	37.7 ± 0.1 (*n* = 57)**	37.1 ± 0.1 (*n* = 140)**	37.1 ± 0.1 (*n* = 48)**	**< 0.0001**	**0.0001**
**Laboratory values at presentation**						
CRP (mg/dl) Threshold ≤5	6.7 ± 0.8 (*n =* 72)**	7.5 ± 0.9 (*n =* 61)**	4.7 ± 0.6 (*n =* 147)**	8.0 ± 1.2 (*n =* 50)**	**0.003**	0.808
Leukocyte counts (G/l) 4.0–10.4 (w) 3.9–9.8 (m)	7.4 ± 0.5 (*n* = 72)**	7.6 ± 0.5 (*n =* 61)**	9.3 ± 0.3 (*n =* 147)**	10.1 ± 0.6 (*n =* 50)**	**< 0.0001**	**0.0003**
LDH (U/) Threshold ≤249	370.0 ± 24.7 (*n =* 68)**	386.3 ± 27.1 (*n =* 60)**	303.5 ± 18.6 (*n* = 135)**	310.4 ± 17.5 (*n =* 50)**	**< 0.0001**	**0.012**
IL-6 (pg/ml) Threshold ≤5.9	104.7 ± 36.5 (*n =* 61)**	113.3 ± 39.6 (*n =* 56)**	177.5 ± 52.9 (*n =* 105)**	279.6 ± 118.9 (*n* = 39)**	0.401	0.297
D-dimer (μg/ml) Threshold ≤5	2.4 ± 0.8 (*n =* 61)**	2.6 ± 0.9 (*n* = 53)**	3.0 ± 0.5 (*n* = 102)**	3.9 ± 1.0 (*n* = 38)**	0.924	0.462
**Result of RT-PCR**						
Positive	72 (100.0)	61 (100.0)	–	–		
By initial test	69 (95.8)	58 (95.1)	–	–		
Several tests necessary for detection	3 (4.2)	3 (4.9)				
By second test	1 (1.4)	1 (1.6)	–	–		
By third test	1 (1.4)	1 (1.6)	–	–		
By fourth test	1 (1.4)	1 (1.6)	–	–		
Negative	–	–	147 (100.0)	50 (100.0)		
**Radiological imaging at presentation**						
**CXR**	15 (20.8)	11 (18.0)	36 (24.5)	7 (14.0)		
Pneumonic features						
Present (score 2)	8 (53.3)	8 (72.7)	6 (16.7)	4 (57.1)		
Possible (score 1)	3 (20.0)	2 (18.2)	7 (19.4)	3 (42.9)		
Absent (score 0)	4 (26.7)	1 (9.1)	23 (63.9)	0 (0.0)		
**Chest CT**	69 (95.8)	61 (100.0)	120 (81.6)	50 (100.0)		
Pneumonic features						
Unilateral		5 (8.2)		21 (42.0)		**< 0.0001**
Bilateral		56 (91.8)		29 (58.0)		**< 0.0001**
Affected lung lobes (mean ± SEM)		4.3 ± 0.1		2.6 ± 0.2		**< 0.0001**
Presence of GGOs		61 (100.0)		48 (96.0)		0.201
Presence of Consolidations		38 (62.3)		35 (70.0)		0.427
COVID-19 features						
Typical (score 2)	45 (65.2)	45 (73.8)	1 (0.8)	1 (2.0)		
Possible (score 1)	12 (17.4)	12 (19.7)	13 (10.8)	13 (26.0)		
Not typical (score 0)	12 (17.4)	4 (6.6)	106 (88.3)	36 (72.0)		

^a^based on the result in RT-PCR for SARS-CoV-2

### Radiological characteristics

Radiological imaging was performed in all patients: 30 (13.7%) patients only received CXR, 164 (74.9%) a thoracic CT scan and 25 (11.4%) both.

In CT examination 111 (58.7% of CT scans) patients had evidence of abnormal findings compatible with pneumonia, 61 (55.0%) of them were tested positive for SARS-CoV-2 in RT-PCR. 50 (45.0%) patients showed pneumonic findings due to another cause with a negative result in RT-PCR for SARS-CoV-2. In comparison, bilateral pneumonia was more common (91.8% vs. 58.0%; *p* < 0.0001) and more lung lobes were affected (4.3 ± 0.1 vs. 2.6 ± 0.2; *p <* 0.0001) in COVID-19 patients than in patients with pneumonia of other causes. There were no significant differences in the presence of ground glass opacities or consolidations between COVID-19 positive and negative patients with pneumonia.

Among COVID-19 patients with pneumonic features (*n* = 61), the abnormal CT findings were evaluated as highly suspicious for COVID-19 (score 2) in 45 (73.8%), as possible (score 1) in 12 (19.7%) and as not typical for COVID-19 (score 0) in four patients (6.6%). At the same time, in patients without COVID-19 the radiological readers defined the pneumonic findings in chest CT as not typical (score 0) in 36 patients (72.0%), as possible (score 1) in 13 (26.0%) and as highly suspicious for COVID-19 (score 2) in one case (2.0%). A total of 78 (41.3% of CT scans) patients showed no pneumatic features in chest CT, 8 (10.3%) of them with positive and 70 (89.7%) with negative RT-PCR for SARS-CoV-2.

Among patients with confirmed COVID-19 by RT-PCR and findings highly suspicious for COVID-19 (score 2) in chest CT, there were ten patients who also received CXR on the same day as CT scan. In all of them the abnormal CT findings due to COVID-19 pneumonia were also detected (*n* = 8, score 2) or at least suspected (*n* = 2, score 1) in CXR.

A detailed overview of initial radiological imaging features is illustrated in Table 2.

### Performance of RT-PCR and chest CT in detecting patients requiring sensitive isolation

Among the patients with a RT-PCR and CT scan (*n* = 189), 83 (43.9%) required a sensitive isolation due to an overall positive result in RT-PCR and/or a positive result for COVID-19 in a sensitive CT reading (score 1 and 2). The sensitivity in the detection of these patients was 79.5% (95% CI 69.6–86.8%) with initial RT-PCR and 90.4% (95% CI 82.1–95.0%) with initial Chest CT. The test performances of RT-PCR and chest CT are reported in Table 3.

**Table 3:** Performance of initial RT-PCR and CT in detecting patients who require isolation

	Results (n)				Test performance (%)				
	TP	TN	FP	FN	Sensitivity [95% CI]	Specificity [95% CI]	PPV [95% CI]	NPV [95% CI]	Accuracy [95% CI]
**Initial PCR**	66	106	–	3	79.5 (66/83) [69.6–86,8]	100 (106/106) [96.5–100]	100 (66/66) [94.5–100]	86.2 (106/123) [79.0–91.2]	91.0 (172/189) [86.1–94.3]
**Initial CT**	75	70	36	8	90.4 (75/83) [82.1–95.0]	66.0 (70/106) [56.6–74.4]	67.6 (75/111) [58.4–75.6]	89.7 (70/78) [81.1–94.7]	76.7 (145/189) [70.2–82.2]

*TP* true positive, *TN* true negative, *FP* false positive, *FN* false negative, *PPV* positive predictive value, *NPV* negative predictive value, *CI* confidence interval.

## Discussion

In the present study we analyzed clinical and diagnostic features including laboratory parameters and radiological imaging features in 219 patients who were presented in the emergency department of our university hospital with signs of a respiratory infection suspicious for COVID-19 within the first 4 weeks the coronavirus pandemic reached our hospital.

The development of patient numbers showed a strong increase in suspected cases and COVID-19 positive patients especially within the first 2 weeks (increase by 67.6 and 92.3%, respectively). While in the following weeks the suspected cases remained at a largely stable but still high level, the number of confirmed COVID-19 patients decreased by over a third. This shows the positive effects of containment measures, but also reveals a nevertheless challenging number of patients for whom optimal management is crucial. In everyday clinical practice, efficient strategies must therefore be developed to sensitively detect COVID-19 patients among the high number of suspicious cases, using available resources efficiently. Typical characteristics of COVID-19 patients can be helpful in an effort to cope with the limited health care capacities as efficiently as possible. It has already been shown that COVID-19 patients have typical characteristics regarding clinical symptoms, laboratory values and radiological findings. However, these studies mainly analyzed patients with confirmed COVID-19 [5–9, 11, 21]. Only a few studies analyzed a cohort of suspected COVID-19 cases as done in our study, but then mostly with a significantly smaller sample size [13, 22, 23] (21 to 38 patients versus 219 in the present study), with a smaller proportion of COVID-19 patients [24] (5% versus 32.9% in the present study) or without analysis of radiological aspects [25]. Thus, our study scrutinizes a patient cohort that has rarely been investigated so far. However, it is particularly this evaluation of a cohort of suspicious cases that allows us to draw conclusions about the clinical setting in emergency departments affected by the COVID-19 pandemic and to demonstrate the potential of typical characteristics of COVID-19 patients compared to patients affected by other respiratory infections for optimal patient management.

Our study was able to identify certain features that were dominant in COVID-19 patients when juxtaposing both groups: Patients with confirmed COVID-19 were more likely to have fever and showed significantly higher values of CRP and LDH. At the same time, leukocyte counts were significantly lower in COVID-19 patients than in patients with respiratory symptoms not caused by COVID-19. Even by correlating laboratory parameters and findings in chest CT concerning pneumonia, our study showed that patients with pneumonia caused by COVID-19 had higher body temperature and LDH values as well as lower leukocyte counts than patients with pneumonia of other origin. This may be a useful adjunct when making a decision regarding the likelihood of COVID-19 infection in cases of disputed abnormal CT findings.

The benefit of comparing those characteristics within a cohort of patients with suspected COVID-19 is also shown by the fact that the results differ when comparing subgroups within confirmed COVID-19 patients. While in the cohort with suspected COVID-19 IL-6 values did not significantly differ between COVID-19 positive and negative patients regardless of the presence of pneumonia, it was precisely this value within the ICU-COVID-19 and Non-ICU-COVID-19 subgroup that was of high significance: COVID-19 patients admitted to an ICU had significantly higher IL-6 values than COVID-19 patients without intensive care treatment. This suggests that IL-6 values may indicate disease severity in COVID-19 patients – as already shown by Herold et al. [27] – but do not allow any conclusion to be drawn about the possibility of COVID-19 infection as a cause of respiratory symptoms. At the same time, leukocyte counts did not significantly differ between ICU-COVID-19 and Non-ICU-COVID-19 subgroup, while this value was particularly relevant between the COVID-19 positive and negative subgroups within patients with respiratory infections. This discrepancy between the different subgroups suggests that transferring the results in cohorts with confirmed COVID-19 - as they have mainly been analyzed so far - to cohorts with suspected COVID-19 and thus to everyday clinical setting in emergency departments is not possible.

Currently, hospitalization and isolation as well as further management of patients is mainly dependent on the results of RT-PCR. Nevertheless, several studies have already demonstrated a lack of sensitivity of RT-PCR [19, 20] and identified CT as an important tool in diagnosing COVID-19. In our study COVID-19 pneumonia differed from pneumonia of other cause only in the extent of the pneumonic features (affecting both lungs and more lobes), but not in the presence of ground glass opacities and consolidations. However, in almost all RT-PCR-positive COVID-19 patients with pneumonic features in CT scan, radiological readers classified those findings as highly suspicious (score 2) or at least possible (score 1) for COVID-19 (57 out of 61; 93.4%). At the same time, in patients with negative RT-PCR and yet pneumonic features in CT scan, these CT findings were evaluated as not typical for COVID-19 (score 0) in most cases (36 out of 50; 72.0%). Thus, COVID-19 could also be detected or excluded with high probability using only CT scan. Nevertheless, in almost one third of those patients with evidence of pneumonia on chest CT but negative RT-PCR for SARS-CoV-2, the abnormal findings were assessed as highly suspicious (score 2) or possible (score 1) for COVID-19 (14 out of 50; 28%). The fact that also in COVID-19 patients confirmed by RT-PCR abnormal CT findings were classified as possible for COVID-19 (score 1) in 19.7% (12 out of 61), shows that CT findings require a sensitive assessment. Even such “possible” COVID-19 cases suspected by CT scan have to be listed as potentially infectious despite negative RT-PCR, at least until further tests provide certainty about the COVID-19 status. In our study, the sensitivity in the detection of patients requiring isolation was higher with initial chest CT than with initial RT-PCR (90.4% vs. 79.5%). Thus, chest CT offers a sensitive selection of patients requiring isolation, monitoring and therapy due to COVID-19, also with an additional detection of possible COVID-19 in almost one third of RT-PCR-negative patients. Even if COVID-19 had been detected by CT scan alone, in case of pneumonic features only 6.6% (4 out of 61) of the patients who actually were diagnosed with COVID-19, determined by RT-PCR, would not have been caught. Therefore, this could also open up possibilities in regions with limited availability of RT-PCR in order to still offer the best possible patient care. Overall, these results suggest that the combination of RT-PCR, clinical and laboratory characteristics as well as chest CT has the potential to provide optimal patient management. In case of doubt, especially in presence of typical symptoms, a supplementary CT scan should be performed even in cases with negative RT-PCR. Especially since our study showed that in 4.2% of patients who have been tested positive overall, the initial RT-PCR showed a negative result, while initial CT scan yet showed pneumonic features in all of them. Only a high sensitivity in diagnosing and isolating COVID-19 patients can prevent a further spread in hospitals among staff and other sick, partly immunocompromised and thus particularly vulnerable patients.

Among COVID-19 patients who received CXR and chest CT, the pneumonic CT features could also be detected or at least suspected in CXR in all ten cases. Therefore, CXR could be a helpful tool, especially in countries and hospitals with limited access to CT scans or RT-PCR. Furthermore, the possibility of detecting COVID-19 pneumonia in CXR can play a major role in COVID-19 patients’ follow-up: In case of an already detected COVID-19 infection, opacities can be monitored by portable CXR in order to save CT resources and prevent contamination and spread due to transport of COVID-19 patients across the hospital [28].

Our study has several limitations such as an ongoing hospitalization of some patients at the time of the submission. Furthermore, the investigated cohort mainly includes patients with a moderate or severe disease or with a high risk for a severe course due to the internal procedure for radiological imaging in patients with suspected COVID-19. This is also shown by the fact that almost all COVID-19 patients were hospitalized, and a comparatively high proportion required intensive care treatment.

## Conclusion

In conclusion, our study showed that despite successful containment measures, the number of suspected COVID-19 cases remains challenging. In comparison with respiratory infections of other causes, COVID-19 patients present certain characteristics regarding clinical symptoms and laboratory values, which can be a useful adjunct in making decisions in a number of cases, even in the assessment of unclear findings in CT scans. Furthermore, the performance of CT scans in patients with respiratory infections suspicious for COVID-19 enables a sensitive selection of those requiring isolation, monitoring and treatment due to COVID-19. Thus, in everyday clinical practice, the combination of RT-PCR, typical clinical and laboratory characteristics as well as thoracic CT is a helpful tool for improved containment and patient management in emergency departments.

## Data Availability

The datasets used and/or analysed during the current study are available from the corresponding author on reasonable request.

## Abbreviations

COVID-19: Coronavirus Disease 19
CRP: C-reactive Protein
CT: Computed Tomography
CXR: Chest Radiograph
GGO: Ground Glass Opacities
ICU: Intensive Care Unit
IL-6: Interleukin-6
LDH: Lactate Dehydrogenase
NPV: Negative Predictive Value
PPV: Positive Predictive Value
RT-PCR: Reverse Transcription Polymerase Chain Reaction
SARS-CoV-2: Severe Acute Respiratory Syndrome Coronavirus 2
